# Wnt signaling pathway inhibitors, sclerostin and DKK-1, correlate with pain and bone pathology in patients with Gaucher disease

**DOI:** 10.3389/fendo.2022.1029130

**Published:** 2022-11-24

**Authors:** Margarita M. Ivanova, Julia Dao, Neil Kasaci, Andrew Friedman, Lauren Noll, Ozlem Goker-Alpan

**Affiliations:** Translational Research, Lysosomal and Rare Disorders Research and Treatment Center, Fairfax, VA, United States

**Keywords:** Gaucher disease, osteoporosis, pain, bone, SOST, DKK-1, Wnt

## Abstract

Patients with Gaucher disease (GD) have progressive bone involvement that clinically presents with debilitating bone pain, structural bone changes, bone marrow infiltration (BMI), Erlenmeyer (EM) flask deformity, and osteoporosis. Pain is referred by the majority of GD patients and continues to persist despite the type of therapy. The pain in GD is described as chronic deep penetrating pain; however, sometimes, patients experience severe acute pain. The source of bone pain is mainly debated as nociceptive pain secondary to bone pathology or neuropathic or inflammatory origins. Osteocytes constitute a significant source of secreted molecules that coordinate bone remodeling. Osteocyte markers, sclerostin (SOST) and Dickkopf-1 (DKK-1), inactivate the canonical Wnt signaling pathway and lead to the inhibition of bone formation. Thus, circulated sclerostin and DKK-1 are potential biomarkers of skeletal abnormalities. This study aimed to assess the circulating levels of sclerostin and DKK-1 in patients with GD and their correlation with clinical bone pathology parameters: pain, bone mineral density (BMD), and EM deformity. Thirty-nine patients with GD were classified into cohorts based on the presence and severity of bone manifestations. The serum levels of sclerostin and DKK-1 were quantified by enzyme-linked immunosorbent assays. The highest level of sclerostin was measured in GD patients with pain, BMI, and EM deformity. The multiparameter analysis demonstrated that 95% of GD patients with pain, BMI, and EM deformity had increased levels of sclerostin. The majority of patients with elevated sclerostin also have osteopenia or osteoporosis. Moreover, circulating sclerostin level increase with age, and GD patients have elevated sclerostin levels when compared with healthy control from the same age group. Pearson’s linear correlation analysis showed a positive correlation between serum DKK-1 and sclerostin in healthy controls and GD patients with normal bone mineral density. However, the balance between sclerostin and DKK-1 waned in GD patients with osteopenia or osteoporosis. In conclusion, the osteocyte marker, sclerostin, when elevated, is associated with bone pain, BMI, and EM flask deformity in GD patients. The altered sclerostin/DKK-1 ratio correlates with the reduction of bone mineral density. These data confirm that the Wnt signaling pathway plays a role in GD-associated bone disease. Sclerostin and bone pain could be used as biomarkers to assess patients with a high risk of BMI and EM flask deformities.

## Introduction

Deficiency of the enzyme glucocerebrosidase (GCase) and accumulation of glucosylceramide (GC) substrate and its product glucosylsphingosine (lyso*-*Gb1) lead to Gaucher disease (GD) (1). GD primarily affects monocyte lineage cells (macrophages), which play essential roles in the immune system and are involved in osteoclast differentiation and osteoclast–osteoblast activities in bone remodeling. Of GD patients, 80% to 95%, including asymptomatic patients, present with different forms of bone involvement, including structural bone changes, debilitating bone pain, and osteoporosis (2–4). Due to abnormal bone remodeling, structural bone pathology includes Erlenmeyer flask deformity, bone modeling abnormality, osteonecrosis, and lytic lesions in GD (2, 5, 6). Bone pain occurs in GD patients even if there have been no fractures or bone disease. Pain remains to be a major complaint in patients with GD and continues to persist despite enzyme replacement therapy (ERT) or substrate reduction therapy (SRT) (7, 8). The pain in GD is described as a chronic deep penetrating pain and tenderness sensation; however, sometimes, patients experience severe acute pain (bone crisis) usually related to ischemic insult. Today, the source of bone pain is mainly debated as nociceptive pain secondary to bone involvement pathology or neuropathic or inflammatory origins (9).

Osteocytes, differentiated from osteoblast, are considered a significant source of secreted molecules that coordinate osteoclast and osteoblast activities in response to physiological changes in the body, including physical, hormonal, age-related, or disease-related status. The canonical Wnt signaling pathway plays an essential role in osteoblast differentiation and bone formation, bone resorption, and bone homeostasis (10–12). Osteocytes secrete sclerostin, which inhibits bone formation by inhibiting the Wnt signaling pathway (13). DKK-1, mainly expressed by pre-osteoblasts and osteoblasts, is also a secreted protein that binds to the LRP6 co-receptor on the cellular membrane and inhibits β-catenin-dependent Wnt signaling (14). Sclerostin and DKK-1 are antagonists of the Wnt/β-catenin signaling pathway due to inhibition of the Wnt pathway. Both molecules initiate the inhibition of bone formation and promote bone resorption. Therefore, sclerostin and DKK-1 neutralizing antibodies (romosozumab and BHQ880) are appealing new strategies for the treatment of inhibition of decreasing bone mineral density and osteoporosis. Both neutralizing antibodies block the inhibition of the Wnt/β-catenin pathway, which plays an essential role in bone formation.

The goal of this study was to assess the plasma level of sclerostin and DKK-1 in patients with GD and correlate it with the degree of bone involvement, including bone pain, bone marrow infiltration, Erlenmeyer (EM) flask deformity, and osteoporosis.

## Materials and methods

### Subjects

Patients with GD ages 18 to 68 years (range 42 ± 15) and 20 healthy controls (range 48 ± 11) participated in the study at a single center Lysosomal and Rare Disorders Research and Treatment center (LDRTC) between 2019 and 2022. GD diagnosis was based on GCase residual activity and *GBA* sequencing analysis. Thirty-eight patients had a known genotype, including 17 patients who were homozygous with N370S. The most common second allele was L444P (n = 12); one patient has L444P/L444P (**Table 1**). Participants were categorized further into three groups based on T- or Z-score of bone mineral density (BMD): the normal cohort, no bone complication (N; an average of T-score 0.03 ± 0.2; Z-scores −0.2, n = 11), the osteopenia cohort (OSN; an average of Z-score −1.07 ± 0.2, T-score −1.6, n = 14), and osteoporosis cohort (OSR; an average of Z-score −2.96 ± 0.8; T-score −2.73 ± 0.4, n = 14). All patients gave a written informed consent form for the collection and analysis of their data. The clinical protocol was approved by ethics committees and data protection agencies at all participating sites (Western Institutional Review Board, WIRB #20131424) and NCT04055831. In addition, healthy controls were recruited under NCT02000310, or plasma was purchased (StemExpress, Folsom, CA, USA).

**Table 1:** Demographic, genotypes, and clinical characteristics of bone disease in patients with GD.

Patient no.	Age(years)	Gender	ERT/SRT duration(years)	Genotype(allele 1/allele 2)	Bone pain	Bone marrow infiltration	EM deformity	Cystic changes	Pathologic fractures/bone surgery	AVN
**No bone complication**										
1	18	F	ERT 5–10	N370S/F213I	Yes	Yes	Yes	No	No/no	No
2	56	F	ERT > 10	N370S/N370S	Yes	Yes	Yes	No	No/no	Yes
3	41	F	SRT	N370S/R463C	No	Yes	Yes	No	No/no	Yes
4	50	F	ERT 5–10	N370S/N370S	No	Yes	Yes	No	No/no	No
5	29	F	SRT	N370S/N370S	No	No	No	No	No/no	No
6	25	F	ERT > 10	L444P/L444P	No	No	No	No	No/no	No
7	37	F	ERT > 10	N370S/W381X	Yes	Yes	No	No	No/no	Yes
8	49	F	ERT > 10	N370S/L444P	Yes	Yes	No	No	No/yes	Yes
9	29	F	ERT 5–10	/L444P	No	No	No	No	No/no	No
10	21	M	SRT	N370S/L444P	No	Yes	No	No	No/no	No
11	33	M	ERT 1–5	R48Q/L444P	Yes	Yes	Yes	No	No/no	No
**Osteopenia (OSN)**										
1	29	F	ERT 1–5	N370S/N370S	No	Yes	Yes	No	No/yes	No
2	61	F	ERT > 10	N370S/N370S	Yes	Yes	Yes	No	Yes/no	Yes
3	32	F	ERT 5–10	N370S/L444P	No	No	No	No	No/no	No
4	52	F	SRT	N370S/N370S	No	Yes	No	No	No/no	No
5	40	F	SRT	N370S/R463C	Yes	Yes	No	No	No/no	No
6	41	F	Naive	N370S/N370S	No	No	No	No	Yes/no	No
7	51	F	SRT 5–10	N370S/L444P	No	No	No	No	No/no	No
8	74	F	SRT 1–5	N370S/N370S	Yes	Yes	Yes	No	No/no	No
9	38	F	ERT > 10	N370S/N370S	Yes	No	No	No	No/no	No
10	61	F	1 year	N/A	Yes	No	No	No	No/no	No
11	65	M	SRT 5–10	N370/N370	Yes	Yes	No	No	No/no	No
12	18	M	Naive	N370S/N370S	No	Yes	No	No	No/no	No
13	37	M	ERT 1–5	N370S/V394L	Yes	No	No	No	Yes	No
14	20	M	ERT > 10	L44P/P266A	No	No	No	No	No/no	No
**Osteoporosis (OSR)**										
1	57	F	SRT 5–10	L444P/R502C	Yes	Yes	Yes	No	No/no	No
2	54	F	ERT > 10	N370S/L444P	Yes	Yes	Yes	No	No/no	Yes
3	20	F	ERT 1–5	N370S/L444P	Yes	No	No	No	No/no	No
4	62	F	SRT	N370S/N370S	Yes	Yes	Yes	Yes	No/no	No
5	30	F	ERT > 10	N370S/N370S	No	Yes	No	No	No/no	No
6	63	F	Naive	N370S/R496H	Yes	Yes	Yes	No	No/no	No
7	44	F	ERT/SRT > 10	L444P/R493C	Yes	Yes	No	No	Yes/yes	No
8	68	F	ERT 5–10	N370S/R463C	Yes	No	Yes	No	No/yes	Yes
9	45	F	ERT 5–10	N370S/N370S	Yes	No	Yes	No	No/yes	No
10	46	F	SRT	N370S/N370S	Yes	No	Yes	No	No	Yes
11	55	M	SRT 0–1	N370S/L444P	Yes	No	No	No	Yes/yes	No
12	36	M	ERT > 10	N370S/Y412X	Yes	Yes	Yes	No	Yes/yes	Yes
13	39	M	ERT 5–10	N370S/N370S	No	No	No	No	No/no	No
14	42	M	SRT 5–10	N370S/N370S	No	Yes	No	No	No/yes	No

GD, Gaucher disease; ERT, enzyme replacement therapy; SRT, substrate reduction therapy; EM, Erlenmeyer; AVN, avascular necrosis.

At enrollment, a detailed medical history that included bone disease characteristics such as bone surgery, bone fracture, bone pain, bone marrow infiltration, EM flask deformity, avascular necrosis (AVN), and osteonecrosis was obtained (**Table 1**). BMD, bone marrow infiltration (BMI), and a summary of skeletal abnormalities from skeletal surveys using X-rays were retrieved by chart review. Bone densitometry and bone marrow involvement were assessed using dual‐energy X‐ray absorptiometry (DXA) and MRI of the lumbar spine, femora, and bilateral hips as routine clinical care. BMD abnormalities, i.e., osteopenia and osteoporosis, were defined with Z- or T-score using the WHO criteria. Information about bone pain was extracted from the patient medical history and by using the Brief Pain Inventory.

### Sample collection

Venous blood samples were collected into EDTA tubes at three different patient visits 6–8 months apart (15). After centrifugation, plasma was collected, aliquoted into small volumes, and stored at −80°C prior to analysis.

### Enzyme-linked immunosorbent assay

The plasma levels of bone markers were measured using commercially available ELISA kits. The sclerostin concentration was measured in 50 µl of plasma using a human sclerostin ELISA kit (Abcam, Cambridge, UK). The concentration of DKK-1 was measured in 100 µl of plasma using a human DKK-1 (Dickkopf-1) ELISA kit (Abcam, Cambridge, UK). The level of Wnt-5a was measured in 50 µl of plasma using a human Wnt-5a ELISA kit, and the level of beta-catenin was measured in 25 µl using a human beta-catenin ELISA (MyBioSource, San Diego, CA, USA). The RANKL was measured in 100 µl of plasma using RANKL ELISA kits (OriGene Technologies Inc., Rockville, MD, USA).

### Statistical analysis

Statistical analysis was performed using Graph Prizm (GraphPad, San Diego, CA, USA). The differences between the two groups were tested by Student’s t-test (unpaired) or F-test. The groups were compared using one-way analysis of variance (ANOVA) followed by Brown–Forsythe, Bartlett’s multiple comparisons, and Kruskal–Wallis tests. The relationships between sclerostin, DKK-1, and RANKL were determined using Pearson’s or Spearman’s correlation technique.

## Results

### Elevated level of sclerostin is associated with pain, bone marrow infiltration, and Erlenmeyer flask deformities in Gaucher disease

**Figure 1:**
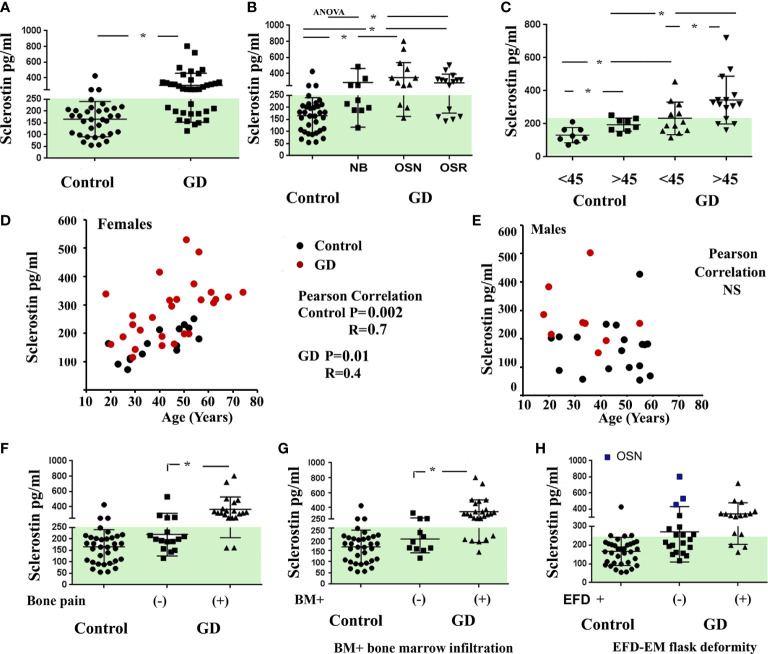
Plasma sclerostin concentrations. **(A)** Sclerostin level (pg/ml), control *vs.* GD. *p < 0.05 unpaired t-test and F-test. **(B)** Sclerostin concentrations in control subjects and GD with no bone complication (N), osteopenia (OSN), and osteoporosis (OSR). p < 0.05; ANOVA, Brown–Forsythe, and Bartlett’s multiple comparison tests. Data are means ± SEM. **(C)** Sclerostin level in female controls and GD patients age-related; cohort divided into two groups before and after 45 years old. *Unpaired t-test p < 0.05. **(D, E)** Scatterplot analysis of the correlation of sclerostin and age in healthy controls and GD female **(D)** and male patients **(E)**. Pearson’s two-tailed correlation. **(F–H)** Sclerostin level in GD patients related to bone pain **(F)**, bone marrow infiltration **(G)**, and EM flask deformity **(H)**. Biomarkers’ normal range is highlighted in green. GD, Gaucher disease; EM, Erlenmeyer.

Sclerostin is a relevant marker of the mature osteocyte pool. Sclerostin inhibits the Wnt/β-catenin signaling pathway by binding the LRP5/6 and Frizzled co-receptors on osteoblasts, reducing bone formation by inhibiting osteoblast differentiation and activity (16). There are no data on the circulated level of sclerostin levels in GD patients. First, we evaluated sclerostin in patients with GD, compared it with the sclerostin of the healthy control cohort, and analyzed its relationship with GD bone disease characteristics. Sclerostin was significantly higher in the GD cohort than in the healthy controls (**Figure 1A**). The majority of GD patients with OSN and OSR showed an elevated level of sclerostin (**Figure 1B**). An increased circulating level of sclerostin has been described as a part of age-related bone formation and bone physiology (17, 18). Therefore, we divided GD female patients into two cohorts according to age: *<*45 and >45 years old. The analysis demonstrated that sclerostin was significantly increased in healthy control and GD patients with age (**Figure 1C**). Moreover, GD patients have elevated sclerostin levels when compared with healthy control from the same age group. Pearson’s correlation analysis showed a significant positive correlation between sclerostin and age in controls and GD female but not male patients (**Figures 1D, E**).

**Figure 2:**
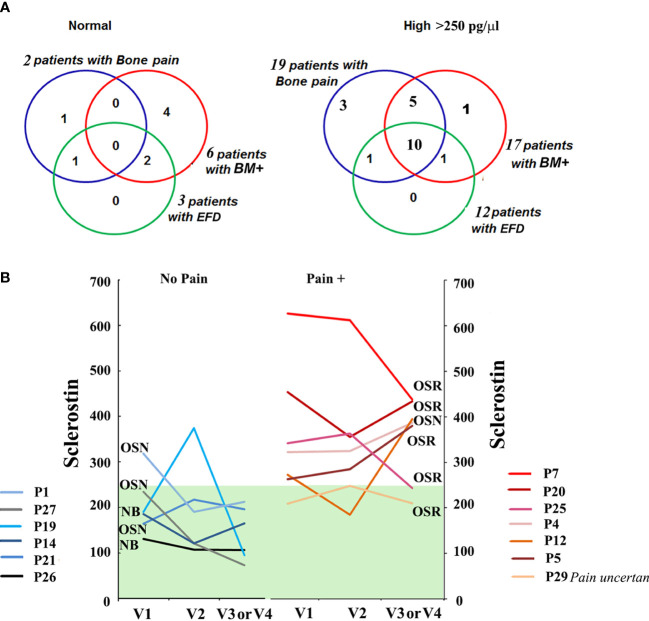
**(A)** The Venn diagrams indicate the number of patients with bone pain, bone marrow infiltration, and EM flask deformity in the GD cohort with a normal range of sclerostin level (left) and high sclerostin level (right). Sclerostin level >250 pg/ml was assessed as an elevated level. **(B)** For GD patients with pain (Pain +) (n = 7, patient P29 described the pain as uncertain) and absent pain (n = 6) who had completed three or four visits, plasma sclerostin levels were measured to assess longitudinal dynamics. Three time points are represented for each participant: initial visit, 6–8 months of follow-up visit (V2), and 12–14 months of follow-up visit (V3). EM, Erlenmeyer; GD, Gaucher disease.

Next, we evaluated the correlation between sclerostin and bone pathology parameters. An elevated sclerostin level was associated with bone pain, bone marrow infiltration, and EM flask deformity in GD (**Figures 1F–H**). The multiparameter analysis demonstrated that 10 out of 21 GD patients with elevated sclerostin levels had bone pain, bone marrow infiltration, and EM flask deformity (Venn diagrams, **Figure 2A**). Four patients with bone pain, bone marrow infiltration, and high sclerostin represent the OSN (n = 2) and OSR (n = 2) cohorts. No GD patient with bone pain, bone marrow infiltration, and EM flask deformity had a normal sclerostin level. Our data suggest that high sclerostin together with bone pain could be useful markers to predict the high risk of bone marrow infiltration and EM flask deformity.

Circulating levels of sclerostin were dynamic in GD patients within 18–24 months of monitoring. Patients with a constant normal range of sclerostin remained without pain, two had OSN, and two had “no” bone pathology. One NB patient and one OSN patient showed an elevated sclerostin (SOST) level episode once. The majority of GD patients with pain presented with consistently high levels of sclerostin over 18 months (**Figure 2B**). The patient who mentioned pain as uncertain showed an average sclerostin level and one borderline episode.

### Circulating DKK-1 correlates with sclerostin

DKK-1, similar to sclerostin, is an extracellular inhibitor of the canonical Wnt/β-catenin signaling pathway. Since the relationship between DKK-1 and OSN-OSR progression in GD patients is unknown, we analyzed the DKK-1 in the GD cohorts. The distribution of plasma DKK-1 in healthy controls showed a wide range from 581 to a maximum of 5,539 pg/ml (2,225 ± 243, mean ± SEM).

**Figure 3:**
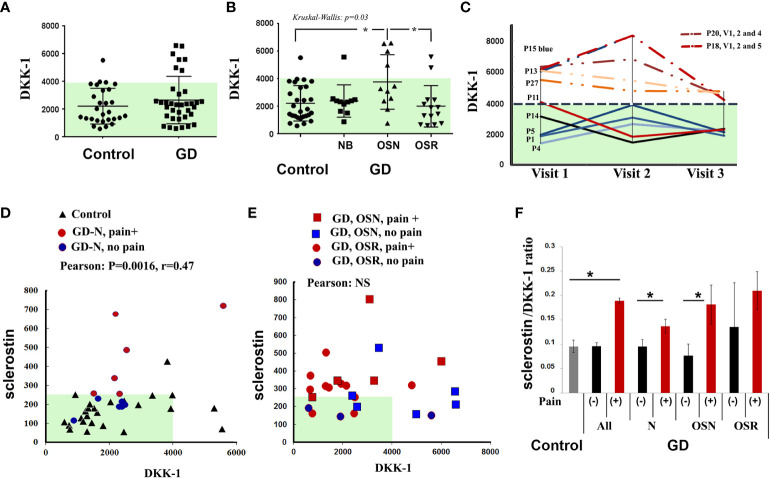
DKK-1 level. **(A)** DKK-1 level, control *vs.* GD. **(B)** DKK-1 concentrations in control subjects and GD with no bone complication (N), osteopenia (OSN), and osteoporosis (OSR). Data are means ± SEM. **(C)** For GD patients with high DKK-1 levels (n = 4) and normal DKK-1 levels (n = 5) who had completed three visits, DKK-1 was measured to assess longitudinal dynamics. Visits are represented for each participant, including an initial visit, 6–8 months of follow-up visit, and a second 12–14 months of follow-up visit, except for P20 visit 4 (18 months) and P18 visit 5 (24 months). **(D)** Scatterplot analysis of correlation of DKK-1 and sclerostin in healthy controls and GD patients with no bone complication, including patients without pain (blue circle) and with pain (red circle). *p < 0.05 Pearson’s and Spearman’s tests, 90%, one-tailed. **(E)** Scatterplot analysis of correlation of DKK-1 and sclerostin in all GD patients with OSN and OSR, including patients without pain (blue color) and with pain (red color). **(G)** Sclerostin/DKK-1 ratio according to the no bone pain or bone pain groups: healthy control group, all GD patients, GD group NB, GD group OSN, and GD-OSRR group. Data are means ± SEM. *p < 0.05 unpaired t-test. DKK-1 and sclerostin measurement pg/ml. GD, Gaucher disease.

Compared with healthy controls, seven patients with GD (20%) had high levels of DKK-1 (**Figure 3A**). The analysis of DKK-1 in the GD cohorts showed an increased level in the OSN cohort compared with the N and OSR cohorts (**Figure 3B**).

In contrast to sclerostin and bone pain, BMI, or EFD–EM relationship, there was no difference in serum DKK-1 among the bone pain/no pain cohorts, BMI, or EFD–EM cohorts (**Supplementary Figure 1**). DKK-1 levels were not changed in GD patients within 18–24 months of monitoring. Patients with a normal range of DKK-1 maintained normal levels, and GD patients with high levels of DKK-1 consistently presented higher levels over 18–24 months (**Figure 3C**).

Because both biomarkers are Wnt/β-catenin inhibitors, we assessed the correlation between sclerostin and DKK-1. After adjusting to normal bone mineral density (N), OSN, and OSR cohorts, Pearson’s and Spearman’s correlation analyses showed a monotonic relationship between sclerostin and DKK-1 in healthy controls only (Spearman’s correlation, r = 0.35, p = 0.04), healthy controls, and GD patients with no bone complications and with/without pain (Pearson’s correlation r = 0.47, p = 0.003116) (**Figure 3D**). However, serum sclerostin and DKK-1 levels did not correlate in the OSN-GD and OSR-GD cohorts together (**Figure 3E**), indicating that increasing the circulation level of sclerostin changes the DKK-1/sclerostin balance in GD patients with OSN and OSR. In addition, significant differences were found in the sclerostin/DKK-1 ratio between no pain and pain GD patients in patients with normal bone mineral density and OSN patients (**Figure 3F**).

### DKK-1, not sclerostin, correlates with RANKL

**Figure 4:**
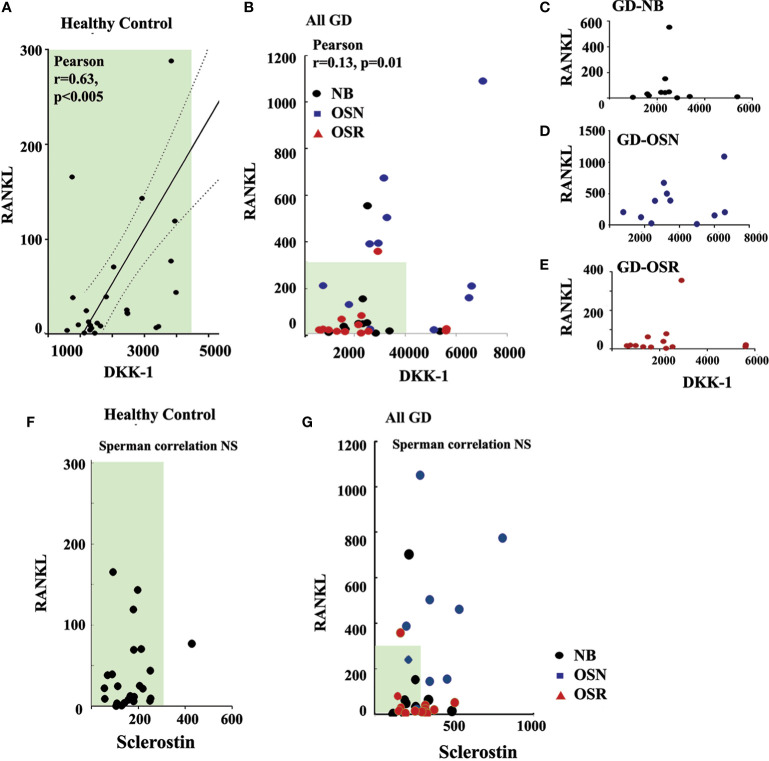
DKK-1 correlates with RANKL in GD patients. **(A)** Scatterplot analysis of correlation of DKK-1 and RANKL in healthy controls. **(B)** Scatterplot analysis of correlation of DKK-1 and RANKL in GD patients. 95%, two-tailed. **(C)** Scatterplot analysis of correlation of DKK-1 and RANKL in GD patients without bone complication (GD-NB). **(D, E)** Scatterplot analysis of correlation of DKK-1 and RANKL in GD patients with OSN) **(E)** and OSR **(E)**. **(F)** Correlation of sclerostin and RANKL in healthy control. **(G)** Sclerostin and RANKL correlation in GD. DKK-1 and sclerostin measurement pg/ml. GD, Gaucher disease; OSN, osteopenia; OSR, osteoporosis.

Because DKK-1 could enhance RANKL through inhibition of the Wnt/β-catenin pathway, and plasma RANKL was elevated in GD patients with OSN (15), we further investigated the correlation between RANKL and DKK-1. Previously, we demonstrated that high RANKL and RANKL/OPG in our cohort correlated with osteopenia (15). Therefore, we used RANKL data from our previous study and correlated them with DKK-1 and sclerostin. Pearson’s and Spearman’s correlation analyses demonstrated a statistically significant positive correlation between serum DKK-1 and RANKL in the healthy control group (Pearson’s two-tailed correlation analysis r = 0.63, p = 0.005, and Spearman’s r correlation analysis r = 0.47, p = 0.013) (**Figure 4A**). Additionally, Pearson’s and Spearman’s linear correlation analyses confirmed a positive correlation between DKK-1 and RANKL in all GD patients (Pearson’s p = 0.0121, r = 0.14) (**Figure 4B**). However, DKK-1 and RANKL levels did not correlate in the NB, OSN, and OSR GD cohorts, possibly due to insufficient sample size (**Figures 4C–E**). In contrast to DKK-1, RANKL and sclerostin did not correlate in the control or GD cohorts (**Figures 4F, G**).

### Elevation of SOST and DKK-1 does not impact the circulating beta-catenin and Wnt5a

Next, we measured β-catenin and Wnt5a levels because Wnt/β-catenin canonical and Wnt5a non-canonical Wnt signaling pathways enhance osteoclastogenesis, while circulating sclerostin and DKK-1 inhibit the Wnt signaling pathway (19). Because β-catenin is a central component of the Wnt pathway, and plasma β-catenin has been proposed as a diagnostic biomarker for postmenopausal women (20, 21), we hypothesized that secreted β-catenin could correlate with osteoporosis in GD. Moreover, several publications mention a negative association between plasma β-catenin and sclerostin or DKK-1 (20, 21). However, there was no significant difference between patients and controls for serum β-catenin levels (control cohort 9.4 ± 4.2 and GD cohort 10.0 ± 5.8). Wnt5a is a Wnt non-canonical ligand that promotes osteoblast differentiation and RANKL-induced osteoclast formation (19). There was no difference between GD patients and healthy controls for circulated Wnt5a levels (control cohort 0.4 ± 0.34 and GD cohort 0.5 ± 0.38).

## Discussion

In the current study, we demonstrated two important findings: 1) an elevated level of sclerostin is associated with pain, bone marrow infiltration, and EM flask deformity in GD; 2) increased circulating sclerostin changes the DKK-1/SOST balance.

Skeletons disorders are often accompanied by bone pain.

Bone diseases associated with GD have a complex of accumulated events/etiologies including abnormal bone development presenting as vertebral remodeling defects, modeling abnormalities of the long bones, and the radiologic hallmark for GD called EM flask deformity (22, 23). Bone destruction can occur due to osteonecrosis, and cystic/lytic lesions are observed with or without AVN. Pathologic fractures occur due to a reduction of BMD, which can start even in teenage years leading, to early osteoporosis in female and male patients with GD (24). Pain is one of GD’s prime and debilitating symptoms, often associated with other structural skeletal involvement (9, 22).

A Brief Pain Inventory analysis in our cohort revealed that 45% of GD patients with normal mineral bone density, 45% with osteopenia, and 78% of patients with osteoporosis report chronic pain. These results are commensurate with the literature that 27%–63% of GD patients have a history of pain (3, 8, 15, 25). Despite the fact that pain is one of the common GD symptoms, the source of pain is still debatable. It has been considered that pain is the result of skeletal involvement, but the pain is described even in the absence of bone disease without a clear explanation (9, 15). The source of pain could be chronic inflammation and/or structural damage to the peripheral nervous system (9). The deterioration of the nerve may present as a result of excessive osteoclast activity that generates a low pH during bone resorption and stimulates acid-sensing ion channels of nerve fibers that innervate bone (26). Because increased osteoclast number and activity have been demonstrated in GD models and patients (15, 25, 27, 28), induced acid-sensing ion channels (acidosis) can play a role in driving bone pain.

Our study’s main conclusion is that elevated sclerostin in plasma is correlated with pain in GD patients. This observation may unravel the association between the role of osteocytes in bone remodeling and bone pain.

If elevated sclerostin correlates with bone marrow infiltration in GD, could the pain be a signal of bone marrow infiltration or predictive of EM flask deformity? For example, bone pain occurs in leukemia patients with the infiltration of white cells into bone marrow (29, 30). The bone marrow is innervated by sensory and sympathetic nerve fibers and nerve fibers involved in the transmission and modulation of bone pain (26). Infiltration of Gaucher cells in the bone marrow may lead to the thinning of the cortex with resultant pain, osteonecrosis, and lytic lesions (7, 31). In addition, BMI induces abnormal bone remodeling. Thus, the remodeling of the distal femora, Erlenmeyer flask deformity, is a common radiological finding in patients with Gaucher disease (31–33). Erlenmeyer flask deformity implies the onset of disease activity in childhood when the skeleton is developing. This deformity, resulting from defective bone modeling at the meta-diaphyseal region, leads to straight uncarved di-metaphyseal borders and cortical thinning (33). The cellular aspects of abnormal bone remodeling that lead to EM flask deformity are not fully understood; however, several studies discuss that osteoclast impairment could be implicated in EM deformity (34).

**Figure 5:**
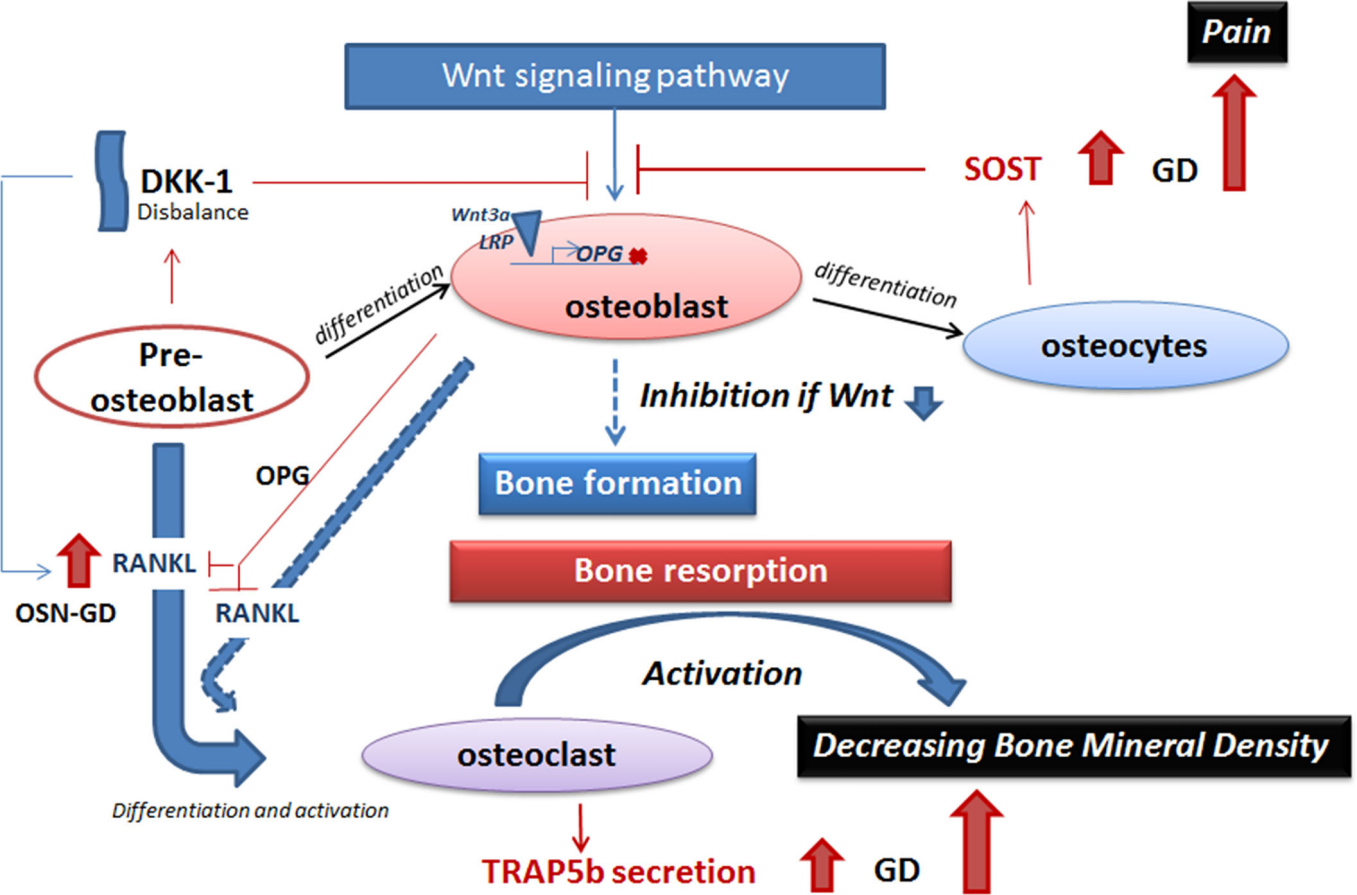
A model of inhibition of the Wnt signaling pathway in GD. The balance between bone formation and bone resorption is controlled by Wnt signaling pathway (activation of bone formation), sclerostin and DKK-1 (inhibition of bone formation), and the RANKL/OPG pathway (osteoclast activation). Elevation of secreted sclerostin or DKK-1 leads to inhibition of Wnt signaling pathway in GD. Sclerostin prevents activation of binding of Wnt 3a and LRP on the cellular membrane and, as a result, inhibits the expression of genes that stimulate bone formation, for example, RANKL inhibitor—OPG. RANKL, expressed by pre-osteoblasts and osteoblasts, promotes osteoclast maturation. Activation of osteoclasts initiates bone resorption. Elevated TRAP5b in GD plasma is the biomarker of osteoclast activity and activation of bone resorption. Activation of bone resorption with inhibition of bone formation leads to decreasing bone mineral density in GD. GD, Gaucher disease.

The dynamic signaling communication between osteoclast, osteoblast, and osteocytes controls bone remodeling. Osteocytes coordinate osteoclast and osteoblast activity, acting as endocrine elements by secreting hormone-like mediators that affect bone cell function and respond to mechanical stimulation on bones (35). One of these mediators is sclerostin. Sclerostin is a cysteine-knot glycoprotein that is predominantly expressed in the bone by osteocytes and less by chondrocytes. Sclerostin regulates bone formation by inhibiting osteoblast–osteocyte differentiation, decreasing bone matrix formation, promoting osteoblast apoptosis, and maintaining bone-lining cells in an inactive state (**Figure 5**) (36–39). Regarding sclerostin as a biomarker for bones, high levels of sclerostin have been detected in patients with thalassemia-associated osteoporosis (40) and abnormal bone remodeling in myeloma (41).

One of the crucial pathways in the skeletal system, the Wnt pathway, is involved in various processes, including the differentiation, proliferation, and synthesis of bone matrix by osteoblasts and differentiation of osteoclasts (12, 42). The Wnt pathway includes the canonical signaling pathway (β-catenin dependent) and the non-canonical or β-catenin independent pathway. β-Catenin is required to activate mesenchymal cells, pre-osteoblasts, to differentiate into osteoblasts and maintain osteocyte viability. Decreased β-catenin activity and inhibition of osteoblast differentiation in GD were previously reported (43, 44). Moreover, several studies on induced pluripotent stem cells (iPSCs) derived from GD patients with null GCase enzyme activity demonstrated defects in the Wnt signaling pathway and increased sclerostin expression (44–46). Interestingly, the treatment of GD iPSC-derived osteoblasts with recombinant GCase enzyme did normalize sclerostin levels (46). One possible mechanism of sclerostin elevation in GD is that functional lysosome is essential for sclerostin degradation (46). However, in GD, accumulation of GC in lysosomes leads to impaired autophagy-lysosomal function, activating the endoplasmic reticulum (ER)-associated degradation pathway and the unfolded protein response (47–52). Thus, sclerostin degradation may be compromised in GD. An additional mechanism of sclerostin elevation may be directly affected by chronic inflammation in GD. For example, a pro-inflammatory cytokine, TNF-alpha, induced the sclerostin expression in osteoblast and osteocyte cells (53–55). Moreover, TNF-alpha contributes to osteoporosis, promoting RANKL-induced osteoclast formation (56, 57). In GD, the elevation of TNF-alpha is associated with macrophage and T-cell activations (58–60).

Dual inhibition of the Wnt signaling pathway by two secreted molecules, sclerostin and DKK-1, demonstrates that multiple mediators likely regulate bone formation. Sclerostin and DKK-1 inhibit the Wnt pathway by blocking binding between Wnt and cell surface receptors—frizzled (FZD) and lipoprotein receptor-related protein 5/6 (LRP6)—and preventing β-catenin translocation to the nucleus (10, 11, 61, 62) (**Figure 5**). If sclerostin is secreted by osteocytes, the source of secreted DKK-1 is mainly pre-osteoblast, osteoblast, and, to a lesser extent, osteocytes (**Figure 5**). Sclerostin inhibits Wnt signaling in the adult bones and keeps the bone lining cells in a quiescent state as part of routine bone maintenance. DKK-1 plays an essential role during skeletal development, and in adults’ bones, DKK-1 is not highly expressed unless activated (14). The increased DKK-1 levels, especially in GD patients with osteopenia, are probably reflective of osteoblasts’ altered differentiation and activity, similar to elevated RANKL in GD patients with osteopenia (15).

Secretion of sclerostin and DKK-1 must be balanced to maintain the Wnt/β-catenin pathway in a steady state and maintain healthy bone mineral density. We have demonstrated a positive correlation between sclerostin and DKK-1 in healthy controls and GD patients with normal bone mineral density. However, the shifting balance between sclerostin and DKK-1 in favor of sclerostin correlates with decreased BMD. A positive correlation between serum sclerostin and serum DKK-1 in a healthy population has been mentioned earlier (63).

Serum sclerostin levels increase with age and are associated with weakening bone formation and activation of bone resorption (64, 65). Moreover, the decreasing bone mineral density with increasing age correlates with sclerostin, especially in postmenopausal women (17, 18, 65–67). Our study also concurs that serum sclerostin level positively correlates with age in healthy controls and female patients with GD. However, sclerostin plasma level was significantly higher in GD patients when compared with age-matched controls. These observations correspond with the datum that in GD, the structural bone changes, including early onset accelerated bone mineral density loss, are less related to age (8). Given that approximately 6% of men and 21% of women aged 50–84 develop osteoporosis, in GD, abnormal BMD is observed in all ages and both genders and further progresses with age (8, 68). For example, in our cohort, the average age of GD patients with normal BMD was 34 ± 12 years, with osteopenia at 46 ± 17 years and osteoporosis at 47 ± 14 years. Our results showed the lack of age-dependent increase in sclerostin levels in male patients with GD, similar to controls. However, the small sample size for male subjects may have impacted the power of the statistical analysis.

The most common therapies used to treat bone pain are non-steroidal anti-inflammatory drugs (NSAIDs) and opiates. “Bone Pain Inventory” analysis of our GD cohort showed that some of our patients used various medications, including NSAIDs (such as ibuprofen) or acetaminophen, and more severe pain was managed with opiates. Moreover, these therapies do not treat the source of the actual cause but only inhibit pain. The treatment of bone pathology and chronic pain in patients is complicated and often insufficient. Inhibition of osteoclast activity may be a solution to inhibit bone resorption and reduce bone pain. Bisphosphonates and denosumab were developed to treat osteoporosis, and both therapies relieve pain in patients with bone cancer (69). Pharmacological inhibition of sclerostin and DKK-1 by monoclonal antibodies has been explored as a potential therapy for osteoporosis, fracture healing, and other bone disorders (69, 70). Also, anti-sclerostin antibodies improve bone mineral density or fracture healing and may relieve skeletal pain (69, 71, 72).

## Conclusion

Elevated sclerostin is associated with reduced bone mineral density, bone pain, bone marrow infiltration, and EM flask deformity in patients with GD. In addition, the altered sclerostin/DKK-1 ratio correlates with the reduction of bone mineral density. In conclusion, our data confirm that the Wnt signaling pathway plays a role in GD-associated bone disease. However, the potential molecular mechanism requires further exploration to design effective therapies for GD-related bone disease.

## Data availability statement

The original contributions presented in the study are included in the article/**Supplementary Material**. Further inquiries can be directed to the corresponding author.

## Ethics statement

The clinical protocol was approved by ethics committees and data protection agencies at all participating sites (Western Institutional Review Board, WIRB # 20131424) and NCT04055831. The patients/participants provided their written informed consent to participate in this study.

## Author contributions

Conceptualization: MI and OG-A. Methodology: JD, LN, NK, and AF. Validation: MI and JD. Formal analysis: MI and OG-A. Investigation: MI and OG-A. Resources: MI. Data curation: MI and OG-A. Writing: MI. Writing—review and editing: MI, OG-A, and JD. Visualization: MI. Supervision: MI. Project administration: MI. All authors contributed to the article and approved the submitted version.

## Funding

This study received funding from an Investigator-Initiated Award from Shire Pharmaceuticals USA, a member of the Takeda group IISR-2019-104331 and IISR-2020-104392 to MI. The funder was not involved in the study design, collection, analysis, interpretation of data, the writing of this article, or the decision to submit it for publication.

## Acknowledgments

The authors would like to thank the clinic staff, patients, and their families whose support made this study possible.

## Conflict of interest

The authors declare that the research was conducted in the absence of any commercial or financial relationships that could be construed as a potential conflict of interest.

## Publisher’s note

All claims expressed in this article are solely those of the authors and do not necessarily represent those of their affiliated organizations, or those of the publisher, the editors and the reviewers. Any product that may be evaluated in this article, or claim that may be made by its manufacturer, is not guaranteed or endorsed by the publisher.

## Supplementary material

Supplementary Figure 1 Serum DKK-1 was not different between the bone pain/no pain cohorts **(A)** and BM cohorts **(B)**.

## References

[1] Pandey MK Grabowski GA. Immunological cells and functions in gaucher disease. Crit Rev Oncog (2013) 18(3):197–220. doi: 10.1615/CritRevOncog.2013004503 23510064 PMC3661296

[2] Mucci JM Rozenfeld P. Pathogenesis of bone alterations in gaucher disease: The role of immune system. J Immunol Res (2015) 2015:192761. doi: 10.1155/2015/192761 26064996 PMC4433682

[3] Oliveri B Gonzalez DC Rozenfeld P Ferrari E Gutierrez G. Grupo de estudio bone involvement gaucher d. early diagnosis of gaucher disease based on bone symptoms. Medicina (B Aires). (2020) 80(5):487–94. 33048793

[4] Mehta A Kuter DJ Salek SS Belmatoug N Bembi B Bright J. Presenting signs and patient co-variables in gaucher disease: outcome of the gaucher earlier diagnosis consensus (GED-c) Delphi initiative. Intern Med J (2019) 49(5):578–91. doi: 10.1111/imj.14156 PMC6852187 30414226

[5] Ivanova M Limgala RP Changsila E Kamath R Ioanou C Goker-Alpan O. Gaucheromas: When macrophages promote tumor formation and dissemination. Blood Cells Mol Dis (2018) 68:100–105. doi: 10.1016/j.bcmd.2016.10.018 27839983

[6] Grabowski GA Antommaria AHM Kolodny EH Mistry PK. Gaucher disease: Basic and translational science needs for more complete therapy and management. Mol Genet Metab (2021) 132(2):59–75. doi: 10.1016/j.ymgme.2020.12.291 33419694 PMC8809485

[7] Hughes D Mikosch P Belmatoug N Carubbi F Cox T Goker-Alpan O. Gaucher disease in bone: From pathophysiology to practice. J Bone Miner Res (2019) 34(6):996–1013. doi: 10.1002/jbmr.3734 31233632 PMC6852006

[8] Goker-Alpan O. Therapeutic approaches to bone pathology in gaucher disease: past, present and future. Mol Genet Metab (2011) 104(4):438–47. doi: 10.1016/j.ymgme.2011.08.004 21889384

[9] Devigili G De Filippo M Ciana G Dardis A Lettieri C Rinaldo S. Chronic pain in gaucher disease: skeletal or neuropathic origin? Orphanet J Rare Dis (2017) 12(1):148. doi: 10.1186/s13023-017-0700-7 28859662 PMC5580212

[10] Mao B Wu W Davidson G Marhold J Li M Mechler BM. Kremen proteins are dickkopf receptors that regulate wnt/beta-catenin signalling. Nature (2002) 417(6889):664–7. doi: 10.1038/nature756 12050670

[11] Li X Zhang Y Kang H Liu W Liu P Zhang J. Sclerostin binds to LRP5/6 and antagonizes canonical wnt signaling. J Biol Chem (2005) 280(20):19883–7. doi: 10.1074/jbc.M413274200 15778503

[12] Glass DA 2nd Karsenty G. Molecular bases of the regulation of bone remodeling by the canonical wnt signaling pathway. Curr Top Dev Biol (2006) 73:43–84. doi: 10.1016/S0070-2153(05)73002-7 16782455

[13] Delgado-Calle J Sato AY Bellido T. Role and mechanism of action of sclerostin in bone. Bone (2017) 96:29–37. doi: 10.1016/j.bone.2016.10.007 27742498 PMC5328835

[14] Pinzone JJ Hall BM Thudi NK Vonau M Qiang YW Rosol TJ. The role of dickkopf-1 in bone development, homeostasis, and disease. Blood (2009) 113(3):517–25. doi: 10.1182/blood-2008-03-145169 PMC2628360 18687985

[15] Ivanova M Dao J Noll L Fikry J Goker-Alpan O. TRAP5b and RANKL/OPG predict bone pathology in patients with gaucher disease. J Clin Med (2021) 10(10). doi: 10.3390/jcm10102217 PMC8160801 34065531

[16] Lewiecki EM. Role of sclerostin in bone and cartilage and its potential as a therapeutic target in bone diseases. Ther Adv Musculoskelet Dis (2014) 6(2):48–57. doi: 10.1177/1759720X13510479 24688605 PMC3956136

[17] Hay E Bouaziz W Funck-Brentano T Cohen-Solal M. Sclerostin and bone aging: A mini-review. Gerontology (2016) 62(6):618–23. doi: 10.1159/000446278 27177738

[18] Modder UI Hoey KA Amin S McCready LK Achenbach SJ Riggs BL. Relation of age, gender, and bone mass to circulating sclerostin levels in women and men. J Bone Miner Res (2011) 26(2):373–9. doi: 10.1002/jbmr.217 PMC3179347 20721932

[19] Kobayashi Y Uehara S Udagawa N Takahashi N. Regulation of bone metabolism by wnt signals. J Biochem (2016) 159(4):387–92. doi: 10.1093/jb/mvv124 PMC4885935 26711238

[20] Tian J Xu XJ Shen L Yang YP Zhu R Shuai B. Association of serum dkk-1 levels with beta-catenin in patients with postmenopausal osteoporosis. J Huazhong Univ Sci Technolog Med Sci (2015) 35(2):212–8. doi: 10.1007/s11596-015-1413-6 25877354

[21] Gaudio A Privitera F Battaglia K Torrisi V Sidoti MH Pulvirenti I. Sclerostin levels associated with inhibition of the wnt/beta-catenin signaling and reduced bone turnover in type 2 diabetes mellitus. J Clin Endocrinol Metab (2012) 97(10):3744–50. doi: 10.1210/jc.2012-1901 22855334

[22] Kaplan P Andersson HC Kacena KA Yee JD. The clinical and demographic characteristics of nonneuronopathic gaucher disease in 887 children at diagnosis. Arch Pediatr Adolesc Med (2006) 160(6):603–8. doi: 10.1001/archpedi.160.6.603 16754822

[23] Pastores GM Meere PA. Musculoskeletal complications associated with lysosomal storage disorders: Gaucher disease and hurler-scheie syndrome (mucopolysaccharidosis type I). Curr Opin Rheumatol (2005) 17(1):70–8. doi: 10.1097/01.bor.0000147283.40529.13 15604908

[24] Itzchaki M Lebel E Dweck A Patlas M Hadas-Halpern I Zimran A. Orthopedic considerations in gaucher disease since the advent of enzyme replacement therapy. Acta Orthop Scand (2004) 75(6):641–53. doi: 10.1080/00016470410004003 15762253

[25] Reed MC Bauernfreund Y Cunningham N Beaton B Mehta AB Hughes DA. Generation of osteoclasts from type 1 gaucher patients and correlation with clinical and genetic features of disease. Gene (2018) 678:196–206. doi: 10.1016/j.gene.2018.08.045 30099023

[26] Mantyh PW. Mechanisms that drive bone pain across the lifespan. Br J Clin Pharmacol (2019) 85(6):1103–13. doi: 10.1111/bcp.13801 PMC6533434 30357885

[27] Reed MC Schiffer C Heales S Mehta AB Hughes DA. Impact of sphingolipids on osteoblast and osteoclast activity in gaucher disease. Mol Genet Metab (2018) 124(4):278–86. doi: 10.1016/j.ymgme.2018.06.007 29934064

[28] Reed M Baker RJ Mehta AB Hughes DA. Enhanced differentiation of osteoclasts from mononuclear precursors in patients with gaucher disease. Blood Cells Mol Dis (2013) 51(3):185–94. doi: 10.1016/j.bcmd.2013.04.006 23707505

[29] Sakata H Nakao A Matsuda K Yoshie N Yamada T Osako T. Acute leukemia presenting as bone pain with normal white blood cell count. Acute Med Surg (2014) 1(4):249. doi: 10.1002/ams2.46 29930859 PMC5997232

[30] Maman E Steinberg DM Stark B Izraeli S Wientroub S. Acute lymphoblastic leukemia in children: correlation of musculoskeletal manifestations and immunophenotypes. J Child Orthop (2007) 1(1):63–8. doi: 10.1007/s11832-007-0013-9 PMC2656700 19308508

[31] Linari S Castaman G. Clinical manifestations and management of gaucher disease. Clin cases Miner Bone Metab (2015) 12(2):157–64. doi: 10.11138/ccmbm/2015.12.2.157 PMC4625773 26604942

[32] Wenstrup RJ Roca-Espiau M Weinreb NJ Bembi B. Skeletal aspects of gaucher disease: a review. Br J Radiol (2002) 75(Suppl 1):A2–12. doi: 10.1259/bjr.75.suppl_1.750002 12036828

[33] Faden MA Krakow D Ezgu F Rimoin DL Lachman RS. The Erlenmeyer flask bone deformity in the skeletal dysplasias. Am J Med Genet A. (2009) 149A(6):1334–45. doi: 10.1002/ajmg.a.32253 PMC2836257 19444897

[34] Adusumilli G Kaggie JD D'Amore S Cox TM Deegan P MacKay JW. Improving the quantitative classification of Erlenmeyer flask deformities. Skeletal Radiol (2021) 50(2):361–9. doi: 10.1007/s00256-020-03561-2 PMC7736022 32734372

[35] Gerosa L Lombardi G. Bone-to-Brain: A round trip in the adaptation to mechanical stimuli. Front Physiol (2021) 12:623893. doi: 10.3389/fphys.2021.623893 33995117 PMC8120436

[36] Sapir-Koren R Livshits G. Osteocyte control of bone remodeling: is sclerostin a key molecular coordinator of the balanced bone resorption-formation cycles? Osteoporos Int (2014) 25(12):2685–700. doi: 10.1007/s00198-014-2808-0 25030653

[37] Tu X Rhee Y Condon KW Bivi N Allen MR Dwyer D. Sost downregulation and local wnt signaling are required for the osteogenic response to mechanical loading. Bone (2012) 50(1):209–17. doi: 10.1016/j.bone.2011.10.025 PMC3246572 22075208

[38] Kitaura H Marahleh A Ohori F Noguchi T Shen WR Qi J. Osteocyte-related cytokines regulate osteoclast formation and bone resorption. Int J Mol Sci (2020) 21(14). doi: 10.3390/ijms21145169 PMC7404053 32708317

[39] Holdsworth G Roberts SJ Ke HZ. Novel actions of sclerostin on bone. J Mol Endocrinol (2019) 62(2):R167–R85. doi: 10.1530/JME-18-0176 30532996

[40] Voskaridou E Christoulas D Plata E Bratengeier C Anastasilakis AD Komninaka V. High circulating sclerostin is present in patients with thalassemia-associated osteoporosis and correlates with bone mineral density. Horm Metab Res (2012) 44(12):909–13. doi: 10.1055/s-0032-1312618 22581647

[41] Terpos E Christoulas D Katodritou E Bratengeier C Gkotzamanidou M Michalis E. Elevated circulating sclerostin correlates with advanced disease features and abnormal bone remodeling in symptomatic myeloma: reduction post-bortezomib monotherapy. Int J Cancer (2012) 131(6):1466–71. doi: 10.1002/ijc.27342 22052418

[42] Bonewald LF Johnson ML. Osteocytes, mechanosensing and wnt signaling. Bone (2008) 42(4):606–15. doi: 10.1016/j.bone.2007.12.224 PMC2349095 18280232

[43] Panicker LM Srikanth MP Castro-Gomes T Miller D Andrews NW Feldman RA. Gaucher disease iPSC-derived osteoblasts have developmental and lysosomal defects that impair bone matrix deposition. Hum Mol Genet (2018) 27(5):811–22. doi: 10.1093/hmg/ddx442 PMC6454561 29301038

[44] Zancan I Bellesso S Costa R Salvalaio M Stroppiano M Hammond C. Glucocerebrosidase deficiency in zebrafish affects primary bone ossification through increased oxidative stress and reduced wnt/beta-catenin signaling. Hum Mol Genet (2015) 24(5):1280–94. doi: 10.1093/hmg/ddu538 25326392

[45] Awad O Panicker LM Deranieh RM Srikanth MP Brown RA Voit A. Altered differentiation potential of gaucher's disease iPSC neuronal progenitors due to wnt/beta-catenin downregulation. Stem Cell Rep (2017) 9(6):1853–67. doi: 10.1016/j.stemcr.2017.10.029 PMC5785733 29198828

[46] Gould NR Williams KM Joca HC Torre OM Lyons JS Leser JM. Disparate bone anabolic cues activate bone formation by regulating the rapid lysosomal degradation of sclerostin protein. Elife (2021) 10. doi: 10.7554/eLife.64393.sa2 PMC8032393 33779549

[47] Ivanova MM Changsila E Iaonou C Goker-Alpan O. Impaired autophagic and mitochondrial functions are partially restored by ERT in gaucher and fabry diseases. PloS One (2019) 14(1):e0210617. doi: 10.1371/journal.pone.0210617 30633777 PMC6329517

[48] Arevalo NB Lamaizon CM Cavieres VA Burgos PV Alvarez AR Yanez MJ. Neuronopathic gaucher disease: Beyond lysosomal dysfunction. Front Mol Neurosci (2022) 15:934820. doi: 10.3389/fnmol.2022.934820 35992201 PMC9381931

[49] Kinghorn KJ Asghari AM Castillo-Quan JI. The emerging role of autophagic-lysosomal dysfunction in gaucher disease and parkinson's disease. Neural Regener Res (2017) 12(3):380–4. doi: 10.4103/1673-5374.202934 PMC5399707 28469644

[50] Maor G Rencus-Lazar S Filocamo M Steller H Segal D Horowitz M. Unfolded protein response in gaucher disease: from human to drosophila. Orphanet J Rare Dis (2013) 8:140. doi: 10.1186/1750-1172-8-140 24020503 PMC3819655

[51] Bendikov-Bar I Ron I Filocamo M Horowitz M. Characterization of the ERAD process of the L444P mutant glucocerebrosidase variant. Blood Cells Mol Dis (2011) 46(1):4–10. doi: 10.1016/j.bcmd.2010.10.012 21106416

[52] Roh J Subramanian S Weinreb NJ Kartha RV. Gaucher disease - more than just a rare lipid storage disease. J Mol Med (Berl) (2022) 100(4):499–518. doi: 10.1007/s00109-021-02174-z 35066608

[53] Vincent C Findlay DM Welldon KJ Wijenayaka AR Zheng TS Haynes DR. Pro-inflammatory cytokines TNF-related weak inducer of apoptosis (TWEAK) and TNFalpha induce the mitogen-activated protein kinase (MAPK)-dependent expression of sclerostin in human osteoblasts. J Bone Miner Res (2009) 24(8):1434–49. doi: 10.1359/jbmr.090305 19292615

[54] Ohori F Kitaura H Marahleh A Kishikawa A Ogawa S Qi J. Effect of TNF-alpha-Induced sclerostin on osteocytes during orthodontic tooth movement. J Immunol Res (2019) 2019:9716758. doi: 10.1155/2019/9716758 31341915 PMC6612957

[55] Baek K Hwang HR Park HJ Kwon A Qadir AS Ko SH. TNF-alpha upregulates sclerostin expression in obese mice fed a high-fat diet. J Cell Physiol (2014) 229(5):640–50. doi: 10.1002/jcp.24487 24446199

[56] Zha L He L Liang Y Qin H Yu B Chang L. TNF-alpha contributes to postmenopausal osteoporosis by synergistically promoting RANKL-induced osteoclast formation. BioMed Pharmacother (2018) 102:369–74. doi: 10.1016/j.biopha.2018.03.080 29571022

[57] Kitaura H Marahleh A Ohori F Noguchi T Nara Y Pramusita A. Role of the interaction of tumor necrosis factor-alpha and tumor necrosis factor receptors 1 and 2 in bone-related cells. Int J Mol Sci (2022) 23(3). doi: 10.3390/ijms23031481 PMC8835906 35163403

[58] Altarescu G Zimran A Michelakakis H Elstein D. TNF-alpha levels and TNF-alpha gene polymorphism in type I gaucher disease. Cytokine (2005) 31(2):149–52. doi: 10.1016/j.cyto.2005.03.006 15919211

[59] Michelakakis H Spanou C Kondyli A Dimitriou E Van Weely S Hollak CE. Plasma tumor necrosis factor-a (TNF-a) levels in gaucher disease. Biochim Biophys Acta (1996) 1317(3):219–22. doi: 10.1016/S0925-4439(96)00056-7 8988238

[60] Mucci JM Scian R De Francesco PN Garcia FS Ceci R Fossati CA. Induction of osteoclastogenesis in an *in vitro* model of gaucher disease is mediated by T cells *via* TNF-alpha. Gene (2012) 509(1):51–9. doi: 10.1016/j.gene.2012.07.071 23010424

[61] Maupin KA Droscha CJ Williams BO. A comprehensive overview of skeletal phenotypes associated with alterations in wnt/beta-catenin signaling in humans and mice. Bone Res (2013) 1(1):27–71. doi: 10.4248/BR201301004 26273492 PMC4472092

[62] Patel S Barkell AM Gupta D Strong SL Bruton S Muskett FW. Structural and functional analysis of dickkopf 4 (Dkk4): New insights into dkk evolution and regulation of wnt signaling by dkk and kremen proteins. J Biol Chem (2018) 293(31):12149–66. doi: 10.1074/jbc.RA118.002918 PMC6078440 29925589

[63] Lu K Li YX Shi TS Yu F Min SC Qiao L. Changes in expression of wnt signaling pathway inhibitors dickkopf-1 and sclerostin before and after total joint arthroplasty. Med (Baltimore) (2017) 96(39):e8082. doi: 10.1097/MD.0000000000008082 PMC5626270 28953627

[64] Coulson J Bagley L Barnouin Y Bradburn S Butler-Browne G Gapeyeva H. Circulating levels of dickkopf-1, osteoprotegerin and sclerostin are higher in old compared with young men and women and positively associated with whole-body bone mineral density in older adults. Osteoporos Int (2017) 28(9):2683–9. doi: 10.1007/s00198-017-4104-2 28585053

[65] Xu Y Gao C He J Gu W Yi C Chen B. Sclerostin and its associations with bone metabolism markers and sex hormones in healthy community-dwelling elderly individuals and adolescents. Front Cell Dev Biol (2020) 8:57. doi: 10.3389/fcell.2020.00057 32117983 PMC7020200

[66] Polyzos SA Anastasilakis AD Bratengeier C Woloszczuk W Papatheodorou A Terpos E. Serum sclerostin levels positively correlate with lumbar spinal bone mineral density in postmenopausal women–the six-month effect of risedronate and teriparatide. Osteoporos Int (2012) 23(3):1171–6. doi: 10.1007/s00198-010-1525-6 21305266

[67] Ueland T Stilgren L Bollerslev J. Bone matrix levels of dickkopf and sclerostin are positively correlated with bone mass and strength in postmenopausal osteoporosis. Int J Mol Sci (2019) 20(12). doi: 10.3390/ijms20122896 PMC6627473 31197079

[68] Masi L Brandi ML. Gaucher disease: the role of the specialist on metabolic bone diseases. Clin cases Miner Bone Metab (2015) 12(2):165–9. doi: 10.11138/ccmbm/2015.12.2.165 PMC4625774 26604943

[69] Fabre S Funck-Brentano T Cohen-Solal M. Anti-sclerostin antibodies in osteoporosis and other bone diseases. J Clin Med (2020) 9(11). doi: 10.3390/jcm9113439 PMC7694131 33114755

[70] Rauner M Taipaleenmaki H Tsourdi E Winter EM. Osteoporosis treatment with anti-sclerostin antibodies-mechanisms of action and clinical application. J Clin Med (2021) 10(4). doi: 10.3390/jcm10040787 PMC7920044 33669283

[71] Mitchell SAT Majuta LA Mantyh PW. New insights in understanding and treating bone fracture pain. Curr Osteoporos Rep (2018) 16(4):325–32. doi: 10.1007/s11914-018-0446-8 PMC6355163 29948820

[72] Frost CO Hansen RR Heegaard AM. Bone pain: current and future treatments. Curr Opin Pharmacol (2016) 28:31–7. doi: 10.1016/j.coph.2016.02.007 26940053

