# Supplementary figures and images for: Wnt signaling pathway inhibitors, sclerostin and DKK-1, correlate with pain and bone pathology in patients with Gaucher disease
Source: Front Endocrinol (Lausanne). 2022 Nov 24;13:1029130. doi: 10.3389/fendo.2022.1029130 (PMC9730525; doi:10.3389/fendo.2022.1029130)

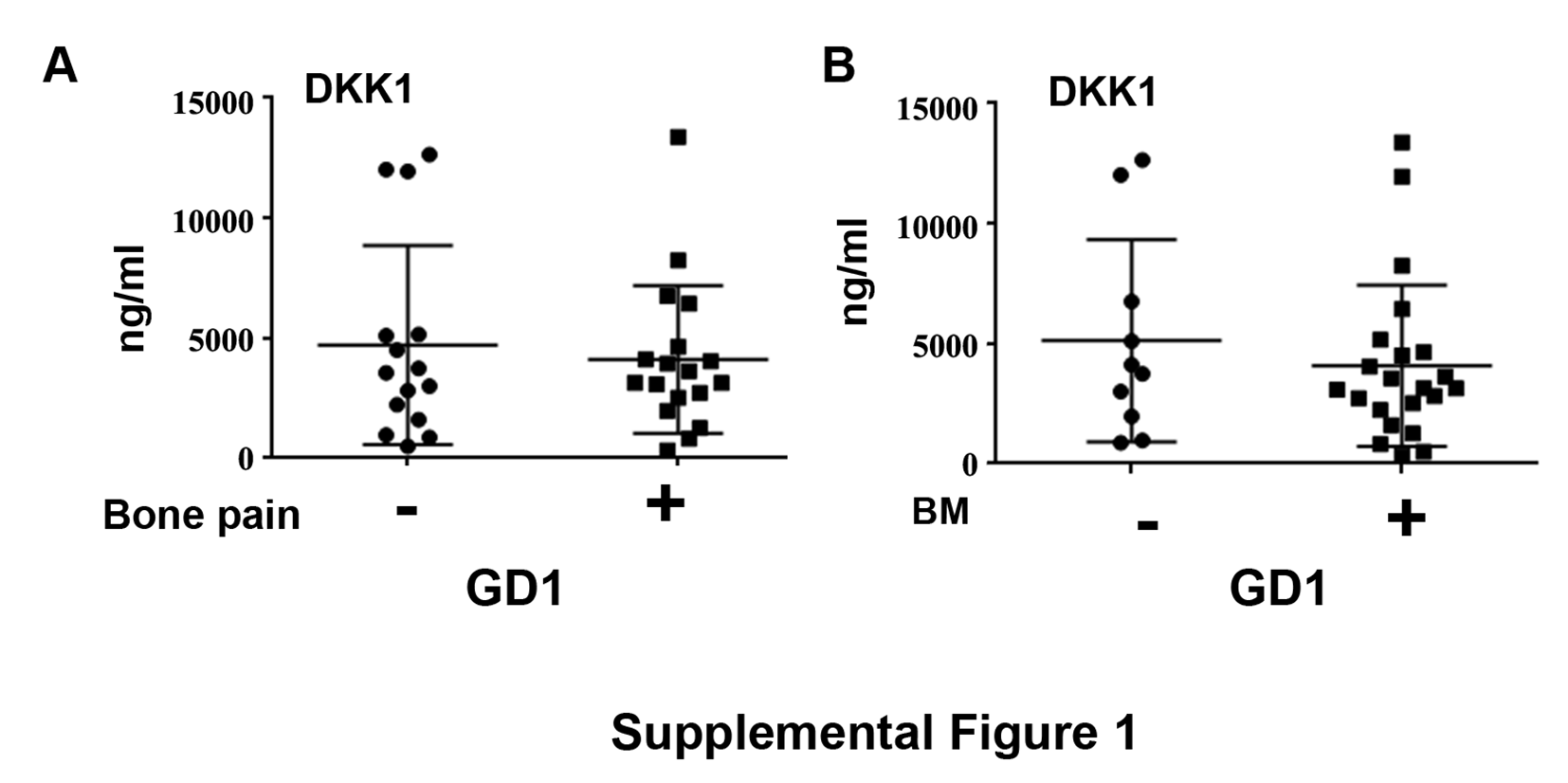

Supplement: Supplementary Figure 1 — Serum DKK-1 was not different between the bone pain/no pain cohorts (A) and BM cohorts (B). [file Image_1.tif]
